# *In vitro* and *in vivo* Studies of Soybean Peptides on Milk Production, Rumen Fermentation, Ruminal Bacterial Community, and Blood Parameters in Lactating Dairy Cows

**DOI:** 10.3389/fvets.2022.911958

**Published:** 2022-08-11

**Authors:** Tian Xie, Fanlin Kong, Wei Wang, Yajing Wang, Hongjian Yang, Zhijun Cao, Shengli Li

**Affiliations:** State Key Laboratory of Animal Nutrition, Beijing Engineering Technology Research Center of Raw Milk Quality and Safety Control, College of Animal Science and Technology, China Agricultural University, Beijing, China

**Keywords:** soybean peptides, degradability, milk production, rumen fermentation, rumen bacteria, dairy cows

## Abstract

Soybean peptides (SPs), a feed additive derived from soybean, exhibit nutritional function and biological activity in monogastric animals, but limited studies have been conducted in dairy cows. Our experiments were conducted to evaluate the effects of SPs on the nutrient degradability of dry matter (DM), crude protein (CP), neutral detergent fiber (NDF), and acid detergent fiber (ADF) *in vitro* and milk production, rumen fermentation and bacterial community, and blood parameters of dairy cows. For *in vitro* experiment, ruminal fluids were collected from three ruminal cannulated Holstein dairy cows. A total of three levels of SPs (0, 0.38, and 1.92 g/kg DM of SPs) were added to the total mixed ration (TMR). Nutrient degradability and fermentation fluid pH were determined at 24 and 48 h using 3.0 g samples of the substrate. Gas production after 48 h was recorded by an automated trace gas recording system using 0.5 g samples of the substrate. The results showed that DM, NDF, ADF (*p* < 0.01), and CP (*p* < 0.05) degradabilities were significantly increased at 1.92 g/kg DM of SPs at 24 h, and asymptotic gas production (*p* = 0.05) was increased at 48 h. For *in vivo* experiment, 110 lactating Holstein cows (209.7 ± 65.2 DIM; 37.2 ± 6.4 kg/d milk yield) were randomly assigned to 0 (control group, CON) or 50 g/head/day SPs (SP-supplemented group). Yields of milk (*p* < 0.05), milk protein (*p* < 0.05), and milk lactose (0.05 < *p* < 0.10) increased on SPs supplementation; however, the milk fat percentage decreased (*p* < 0.05). The concentrations of individual volatile fatty acids (VFAs) (*p* < 0.05) and superoxide dismutase (SOD) (*p* < 0.01) were also increased. Rumen bacterial diversity in SP-supplemented cows was higher (*p* < 0.05). The relative abundances of *Rikenellaceae_RC9_gut_group, Butyrivibrio, Selenomonas*, and *Shuttleworthia* were significantly increased and that of *Coprococcus* was decreased (*p* < 0.05). Our results showed that supplementing 1.92 g/kg DM of SPs could improve the nutrient degradability *in vitro* and 50 g/head/day of SPs could improve milk production and antioxidant ability of dairy cows. The rumen bacterial diversity was also enhanced by SP supplementation.

## Introduction

Soybean is the main ingredient of high-quality proteins used in livestock production. However, it generally contains many protein-type allergens and anti-nutritional factors that affect the performance of monogastric animals and ruminants (1, 2). Proteins can be administered as peptides, which are converted into microbial crude protein (MCP) more efficiently than amino acids (3). Considering intestinal absorption, peptides, especially dipeptides, can be absorbed directly and faster into enterocytes than free amino acids (FAA) (4). Peptides derived from plant proteins could exhibit biological activity both *in vitro* and *in vivo* (5, 6).

Soybean peptides (SPs) can be obtained from *in vitro* enzymatic hydrolysis, microbial fermentation, and gastrointestinal digestion of soybean proteins (7). They usually comprised of 2–10 amino acid (AA) residues (8), and the percentage of soybean oligopeptides with molecular weight <1,000 Da is up to 89.16% when subjected to alcalase hydrolyzation (5). SPs have shown beneficial biological functions in addition to their nutritional value. At present, research involving SPs focuses primarily on their bioactive effects on human health, such as hypertension prevention (9), prebiotic effects on intestinal microbiota (10, 11), intracellular antioxidant activity (12), anticancer activity (13), and immunomodulatory effects (5, 14). The application of SPs as feed additives has emerged in livestock production, and their beneficial effects have been demonstrated in monogastric animals. In broilers, SPs have the potential to stimulate intestinal mucosal immunity, followed by enhanced nutrient digestibility and ideal growth performance (15, 16). In sows, SPs can improve reproductive performance (17).

Ruminants have different digestion and absorption mechanisms than monogastric animals; SPs, especially those smaller than 1 kDa, are rapidly utilized by the rumen microorganisms to synthesize MCP with a balanced amino acid profile, which has high apparent digestibility in the host (18, 19). Both *in vitro* and *in vivo* experiments have shown that SPs display positive effects as feed additives for ruminants. *In vitro* studies have shown that SPs <1 kDa increased dry matter (DM) digestibility at 24 h; at 48 h, neutral detergent fiber (NDF) and acid detergent fiber (ADF) digestibility increased with increasing doses of SPs from 0 to 0.75% (DM basis), and gas production and total volatile fatty acid (TVFA) concentration were also significantly increased (8). However, the digestibility of DM reached its highest when SPs replaced 10% of total nitrogen (N) in a diet containing 46% non-structural carbohydrates in continuous culture, whereas the NDF and ADF digestibilities decreased linearly with SPs addition (20). An *in vivo* study showed that 10 g/d compound SPs significantly improved the milk yield by 3.21 kg/d (21). The growth of rumen cellulolytic bacteria, such as *Butyrivibrio fibrisolvens* and *Succinimonas amylolytica*, was stimulated when SPs were added *in vitro* (8). However, whether the regulation of rumen microorganisms using SPs contributes to the improvement in production performance of dairy cows needs to be further investigated.

To our knowledge, studies on the effects of SP application on the performance of lactating dairy cows are limited. SPs are a mixture of soybean protein hydrolysates, and the inclusion level of SPs varies because of their molecular weight, amino acid composition, and amino acid contents. Si et al. (21) reported that the inclusion of SPs could increase the apparent total tract digestibility (ATTD) of nutrients and immunity of dairy cows to further improve milk yield and milk characteristics. We hypothesized that SPs added at the optimal level could improve the milk production and health status of dairy cows. To test our hypothesis, SPs were introduced into an *in vitro* fermentation system and an *in vivo* experimental. The objective of this study was to determine the effect of optimal level of SPs on the highest nutrient degradability and gas production *in vitro*. Based on the results of the *in vitro* experiment, we aimed to explore the effects of administering the optimal level of SPs to lactating dairy cows on milk production, rumen fermentation, ruminal bacterial community, nutrient digestibility, and blood parameters.

## Materials and Methods

### Ethical Statement

Our experimental procedures were approved by the Institutional Review Board of the China Agricultural University (Protocol number: AW81102202-1-3 and 28.4.2017 of approval). During the experimental period, all animals involved in this study were managed according to the Herd Standard Protocol at the Sunlon Livestock Jinyindao Farm (Daxing County, Beijing, China).

### *In vitro* Experiment

A total of three ruminal cannulated lactating Holstein cows (67 ± 9 DIM; 550 ± 25.4 kg BW; mean ± standard deviation) were used as the ruminal inoculum donors. Cows were fed a basal (control) diet in the *in vivo* experiment (Table 1). Then, 1 h before morning feeding, fresh ruminal fluid was collected and filtered *via* a four-layer cheesecloth into two prewarmed bottles to maintain anaerobic conditions. In the laboratory, the ruminal fluid was warmed in a 39°C water bath.

**Table 1:** Ingredients and chemical composition of total mixed ration (dry matter basis, %).

**Item^**a**^**	**Content**
Ingredient, % of DM	
Alfalfa hay	12.80
Oaten hay	3.54
Alfalfa silage	3.87
Whole corn silage	26.32
Steam-flaked corn	17.03
Corn	12.32
Whole cottonseed	5.46
Extruded soybean meal	4.45
Wheat bran	1.22
Soybean hull	0.45
Distillers dried grains with solubles	1.12
Beet pulp pellet	7.67
Cane molasses	1.14
Limestone	0.31
NaHCO_3_	0.56
NaCl	0.23
Fat powder	0.45
Premix^b^	1.06
Chemical composition, % of DM	
DM, % of fresh-air basis	55.9
NDF	29.3
ADF	18.2
CP	16.7
Starch	24.5
Ash	6.7
Ether extract	5.1
Ca	0.7
P	0.3
NE_L_, (MJ/kg)^c^	7.28

a *DM, dry Matter; NDF, Neutral Detergent Fiber; ADF, Acid Detergent Fiber; CP, Crude Protein; NE_L_: net Energy for Lactation*.

b *One kg Premix Containing 204,881 IU Vitamin A; 23,685 IU Vitamin D; 900 IU Vitamin E*.

c *Estimated From the Nutrient Requirements of Dairy Cattle (22). According to the Nutrient Composition of the Analyzed Ingredients*.

The fresh TMR contained 26 kg dry matter and was mixed in the farm (MVS-16-H self-propelled vertical double auger, TATOMA, Monzón, Spain). Soybean peptides were derived from enzymatic hydrolysis of soybean in powdered form and were provided by TAIDU Biotechnology Group Co., Ltd (Hebei, China). Its AA composition is shown in Table 2. The additive amounts of SPs were based on the experiment of Si et al. (21). SPs were added to the TMR to obtain levels of 0, 0.38, and 1.92 g/kg DM of SPs, and the TMR samples were brought back to the laboratory. A total of three treatments were performed, with six bottles for each treatment at each time point. The three groups of mixed substrates were dried at 60°C for 48 h and ground to pass through a 1-mm mesh with a feedstuff mill (KRT-34; KunJie, Beijing, China). A buffer solution based on Zheng et al. (23) was prepared and continuously flushed with CO_2_ for approximately 30 min until the pH was approximately 6.8. About 3.0 g of TMR was dispensed into each incubation bottle (volume capacity of 300 ml). An incubation medium, containing 75 ml of ruminal fluid and 150 ml of buffer solution, was then added to each bottle and CO_2_ was flushed for approximately 3 s to guarantee anaerobic conditions inside the bottle. A total of 36 bottles were closed with rubber stoppers and maintained in a thermostatic incubator with 18 bottles at the 24- and 48-h time points. Another set of 18 bottles (volume capacity of 120 ml) was prepared similarly but with 0.5 g of substrates and 25 and 50 ml of ruminal fluid and buffer solution, respectively. These bottles were directly connected to an Automated Trace Gas Recording System (AGRS-III, China Agricultural University, Beijing, China) after sealing with parafilm caps. Bottles were incubated in AGRS-III at 39°C for 48 h to record total gas production and gas parameters.

**Table 2:** The composition and content of amino acids in soybean peptides (dry matter basis, %).

**Item**	**Content**
Aspartic acid	4.40
Glutamic acid	6.79
Serine	1.60
Histidine	0.88
Glycine	1.60
Threonine	1.59
Arginine	3.00
Alanine	1.68
Tyrosine	0.92
Cystine	0.57
Valine	1.78
Methionine	0.46
Phenylalanine	1.68
Isoleucine	1.57
Leucine	2.42
Lysine	2.58
Proline	1.97
TAA^a^	35.49

a *TAA, Total Amino Acid*.

After 24 and 48 h of fermentation, the bottles were removed from the incubator. The contents of every two of the six bottles in each treatment were randomly pooled together, poured into one preweighed nylon bag (80 mm × 150 mm size, 42 μm pores), and then washed 5 times manually in cold tap water, followed by drying at 60°C for 48 h in a forced-air oven (Wujiang Zhongda Electrical Technology Co., Ltd., Wujiang, Jiangsu, China). The content of DM, NDF, ADF, and CP of the original substrates and residual samples were determined for nutrient degradability. The fermentation liquid was treated in the same way, and pH was detected using a pH meter (pH B-4 electrode; Shanghai Chemical, Shanghai, China) immediately after fermentation was stopped.

### *In vivo* Experiment

#### Experimental Design and Animal Management

The *in vivo* experiment was performed from 8 March to 15 May 2021. A total of one hundred and ten healthy Holstein cows (209.7 ± 65.2 DIM; 37.2 ± 6.4 kg/d milk yield; mean ± standard deviation) were housed in two separate free-stall barns, with 55 dairy cows in each barn. Holstein dairy cows in the two barns were randomly assigned to one of two isonitrogenous and isoenergetic diets with either 0 (control group, CON) or 50 g/head/day SPs (SP-supplemented group). The amount of 50 g/head/day SP addition was equivalent to 1.92 g/kg DM of SPs in the *in vitro* experiment. Feed ingredients and the chemical composition of the basal (control) diet are shown in Table 1.

The experimental period included 7 days of adaptation and 60 days of sampling and data collection. Dairy cows were fed at 0700, 1400, and 2030 h during the experimental period. SPs were top-dressed daily to the treatment group cows before the morning feeding. Cows were locked in headlock for 30 min after the morning milking to ensure that the SPs were taken by the cows. The diets were prepared every day, and orts were weighed and recorded every morning. The average amounts of feed offered to cows were adjusted to ensure 5–10% refusals. Cows were fed *ad libitum*, and fresh drinking water was available all the time.

#### Milk Production Performance

Cows were milked at 0830, 1500, and 2100 h at the milking parlor during the experimental period. At each milking, milk production was recorded by the DeLaval milking system (DeLaval RTSMU450, A.B. DeLaval, Sweden) to calculate the daily milk yield for each of the 55 cows in the two groups. On days 1, 15, 30, 45, and 60, approximately 50 ml of milk samples for each cow was collected and preserved with potassium dichromate at 4°C. Milk components (milk protein, fat, and lactose content) were analyzed at the DHI Testing Center (Beijing, China). The calculation formula of 3.5% fat-correct milk (3.5% FCM) yield was [milk yield (kg) × 0.4324 + milk fat (kg) × 16.218], and that for the energy-corrected milk (ECM) yield was [milk yield (kg) × 0.3246 + milk fat (kg) × 12.86 + milk protein (kg) × 7.04] [Nutrient Requirements of Dairy Cattle (22)].

#### Dry Matter Intake Calculation and Fecal Sample Analysis

The daily amounts of feed offered to cows in each barn were recorded, and refusals were weighed once before the morning feeding. Weekly diet samples were dried at 60°C for 48 h and grounded using a feedstuff mill (KRT-34; KunJie, Beijing, China) to pass through a 1-mm mesh before chemical analysis. The average dry matter intake (DMI) of each treatment was calculated based on the DM of TMR measured at 105°C.

A total of five cows with milk yield close to the average in each treatment group were selected for fecal sampling. Feces were taken as spot samples three times a day, 8 h apart on days 58, 59, and 60. At each sampling time, approximately 250 g of feces was taken from the rectum of each cow and kept at −20°C. After final sampling, the fecal samples were pooled by cow, and then, 75 ml of 10% tartaric acid was added and dried at 60°C for 48 h for apparent digestibility determination.

DM, CP, calcium (Ca), phosphorus (P), ether extract, and ash concentrations of TMR were analyzed based on the methods of the Association of Official Analytical Chemists (AOAC) (24). The NDF and ADF were analyzed based on Van Soest et al. (25). An ANKOM fiber analyzer (A2000i; American ANKOM, Macedon, NY, USA) was used for NDF and ADF measurements, and heat stable alpha-amylase (ANKOM Technology Co., Ltd., Macedon, NY, USA) was added for NDF analysis. The starch concentration was determined using a commercial assay kit (BioVision, Inc., San Francisco, CA, USA). Starch was hydrolyzed to glucose and tested at 570 nm. The ATTD of the dietary nutrients was determined using acid-insoluble ash (AIA) marker following the description by Hao et al. (26). The nutrient ATTD was calculated using the formula:


(1) $$\begin{array}{l} ATTD\left(\%\right)\;=\;\left[1-\frac{Ad\times Nf}{Af\times Nd}\right]\times \;100 \end{array}$$

where Ad (g/kg) and Af (g/kg) are AIA concentrations in the diet and feces, respectively; and Nd (g/kg) and Nf (g/kg) are concentrations of nutrients in the diets and feces, respectively.

#### Ruminal Fluid Collection and Analysis

The same five cows in each treatment group were selected for ruminal fluid collection. On day 60, the ruminal fluid was collected by an oral gastric tube (Ancitech, Winnipeg, MB, Canada) 2 h after morning feeding. To avoid saliva contamination, the first 50 ml of fluid was discarded, the next 50 ml was collected into a sterile tube, and the rumen pH was measured immediately. Then, the rumen fluid was subdivided into 2-ml freezing tubes and stored at −80°C for 16S rRNA sequencing. The remaining fluid was preserved at −20°C for fermentation parameter analysis.

The rumen fluid was thawed at 4°C for ammonia-N (NH_3_-N), microbial crude protein (MCP), and volatile fatty acid (VFA) concentration analysis. The phenol-sodium hypochlorite colorimetry method (27) and Coomassie brilliant blue colorimetry method (28) were used to measure the NH_3_-N and MCP concentrations, respectively, at 595 nm. VFA concentrations were measured by gas chromatography (TP-2060 system, B. F. TianPu, Beijing, China) (29).

#### Blood Sample Collection and Analysis

The same five cows in each treatment group were used for blood collection *via* the coccygeal vein. Approximately 8 ml of blood was collected using 10-ml evacuated heparin-coated tubes (Vacutainer; Becton, Dickinson and Company, Franklin Lakes, NJ, USA) before the morning feeding on day 60. After centrifugating at 3,500 × g at 4°C for 15 min, the sera were dispensed into 2-ml freezing tubes and stored at −20°C.

Blood colorimetric commercial kits (DiaSys Diagnostics Systems GmbH, Frankfurt, Germany) were used for detecting the concentration of serum insulin (Ins), glucose (Glu), triglyceride (TG), total cholesterol (TC), beta-hydroxybutyric acid (BHBA), non-esterified fatty acid (NEFA), total amino acid (TAA), superoxide dismutase (SOD), catalase (CAT), and glutathione peroxidase (GSH-Px). Bovine ELISA kits (Beijing Laibotairui Technology Development Co., Ltd, China, Nanjing Jiancheng Bioengineering Institute, Nanjing, Jiangsu, China) were used for measuring the concentration of immunoglobulin A (IgA), immunoglobulin G (IgG), and immunoglobulin M (IgM).

#### 16S rRNA Analytical Procedure

Genomic DNA of the microbial community was extracted from the rumen fluid sampled *in vivo* using a Stool DNA kit (Omega Bio-Tek, Norcross, GA, USA). The quality and quantity of DNA were determined using 1% agarose gel electrophoresis. The hypervariable V3–V4 region of the 16S rRNA gene was amplified using primers 338F (5′-ACTCCTACGGGAGGCAGCAG-3′) and 806R (5′- GGACTACHVGGGTWTCTAAT-3′) (30). Polymerase chain reaction (PCR) amplification was performed using the ABI StepOnePlus Real-Time PCR System (Life Technologies, Foster City, CA, USA) following a previous study (26). Amplicons were extracted from 2% agarose gel electrophoresis, purified using the AxyPrep DNA Gel Extraction Kit (Axygen Biosciences, Union City, CA, USA), and quantified using the QuantiFluor™ -ST system (Promega, Madison, WI, USA). Finally, purified amplicons were pooled in equimolar concentrations and pair-end sequenced on an Illumina MiSeq platform (Illumina, Inc., San Diego, CA, USA).

The raw 16S rRNA gene sequencing reads were demultiplexed, quality-filtered using FASTP version 0.20.0 (31), and merged using FLASH version 1.2.7 (32). The criteria of raw read filtering were the same as the study by Hao et al. (26). Thereafter, the primers were completely matched, allowing for a two-nucleotide mismatch. The Quantitative Insights into Ecology (QIIME) program (version 1.9.0) was used to process the raw sequencing data (33). Sequences with 97% similarity were clustered into the same operational taxonomic unit (OTU) using the USTRA-fast sequence analysis program (version 10.0.240) (34). The representative sequences of each OTU were screened for further annotation. The representative sequences were retained with the following criteria: (i) The sequences were ≥5 in at least three samples; (ii) the sum of sequence numbers was ≥20; and (iii) sequence numbers remained the same in accordance with the minimum number of sampling sequence. The ribosomal database project (RDP) classifier was used to assign species according to the Silva bacterial alignment database (35) with a confidence threshold of 0.7 (36). The raw reads were available at the National Center for Biotechnology Information (NCBI) database (BioProject accession ID: 779612, RUN: SRR 18218289 – SRR 18218298, https://www.ncbi.nlm.nih.gov/Traces/study/?page=2&acc=PRJNA779612&o=acc_s%3Aa), (accessed on March 4, 2022).

### Calculations and Statistical Analysis

*In vitro* dry matter degradability was calculated according to the description of Zheng et al. (23) using Excel (Version 2019, Microsoft Corporation, Redmond, WA, USA). The same formula was used to calculate *in vitro* CP, NDF, and ADF degradabilities, replacing DM with the corresponding index. Gas production (GP, ml/g, dry matter basis) and parameters were fitted according to Equation (1) using the non-linear regression (NLIN) procedure in statistical analysis system (SAS) 9.4 (SAS Institute Inc., Cary, NC, USA) (37):


(2) $$\begin{array}{l} GPt\;=\;\frac{A}{1\;+\;{\left(\frac{C}{t}\right)}^{B}} \end{array}$$

where GP_t_ represents the gas production (mL/g DM) at incubation time *t* (h), A represents the theoretical maximum gas production (mL/g DM), B represents the point of inflection on curve parameter, C represents the time (h) when half of the total gas production was reached, and t represents the incubation time. Effects of treatments on the *in vitro* degradability, gas production, and parameters were analyzed using one-way analysis of variance (ANOVA). Tukey's multiple comparison test was used to determine the statistical differences for variables.

For *in vivo* experiment, data were normally distributed. The mean values of yields of milk, protein, fat, and lactose for each one of the 55 cows were calculated every 2 weeks prior to statistical analyses. The milk production performance (milk yield; percentage and yields of milk protein, fat, and lactose) was analyzed using the PROC MIXED procedure in SAS (version 9.4; SAS Institute Inc., Cary, NC, USA), with the fixed effects of diet, time, and diet × time interaction and the random effects of individual animals. Effects of treatments on the rumen fermentation profile, blood parameters, and apparent digestibility were analyzed using one-way analysis of variance (ANOVA).

Chao1, Ace, number of OTUs, and Shannon indices were calculated using QIIME (33). Beta-diversity was measured according to Bray–Curtis distance and binary Jaccard distance and displayed using principal coordinate analysis (PCoA) and hierarchical clustering analysis, respectively. Analysis of similarities (ANOSIM) with 999 permutations was employed to assess the significant differences between CON and SP groups in QIIME (33). The difference in the relative abundance of bacteria between groups was expressed as a percentage and plotted with error bars using STAMP software (38). The predicted functions of rumen bacteria were analyzed through phylogenetic investigations of communities by reconstructing unobserved states (PICRUSt2) software (version 2.2.0). GraphPad Prism 8 (GraphPad Software, San Diego, CA, USA) was used to plot the alpha-diversity indices and the function within the Kyoto Encyclopedia of Genes and Genomes (KEGG) pathways levels 2 and 3. Welch's *t*-test was used to calculate the *p*-value of the alpha-diversity indices, the difference in relative abundance of bacteria, and the predicted functions of rumen bacteria (38).

*p-*Value ≤ 0.01 indicates a highly significant difference, *p* ≤ 0.05 indicates a significant difference, and 0.05 < *p* ≤ 0.10 indicates tendencies.

## Results

### *In vitro* Degradability, Ruminal Fluid pH, and Gas Production

*In vitro* DM, CP, NDF, and ADF degradabilities increased with SPs supplementation at both 24 and 48 h (Table 3). The level of 1.92 g/kg DM of the SP group showed higher DM degradability than the other diet groups (*p* < 0.01) at 24 h. Degradabilities of DM (*p* < 0.01), NDF (*p* < 0.01), and ADF (*p* < 0.01) were significantly increased in the SP-supplemented group than in the control group. After 48 h of incubation, increasing trends were found for the degradability of DM, CP, and ADF (0.05 < *p* ≤ 0.10), except NDF degradability (*p* > 0.05). SP supplementation had no apparent effects on gas production and kinetic parameters B and C (*p* > 0.05). Total gas production was the highest in the group supplemented 1.92 g/kg DM of SPs (*p* > 0.05), and kinetic parameter A in 1.92 g/kg DM of SPs group was significantly larger in than that of the CON group (*p* = 0.05) after 48 h of fermentation. No effects of supplemental SPs on the ruminal fermentation liquid pH were observed (*p* > 0.05).

**Table 3:** Effects of soybean peptides (SPs) on the *in vitro* degradability of DM, CP, NDF, and ADF, ruminal liquid pH, gas production, and kinetic parameters after 24 and 48 h of incubation.

**Variable^**1**^**	**SPs (g/kg DM)**			**SEM**	***P***
	**0**	**0.38**	**1.92**		
Degradability (%)					
DM					
24 h	59.7^c^	63.0^b^	65.2^a^	0.84	<0.01
48 h	64.6	67.1	70.3	1.08	0.07
CP					
24 h	51.4^b^	52.4^ab^	55.9^a^	0.82	0.02
48 h	66.2	66.3	72.5	1.41	0.10
NDF					
24 h	28.2^b^	34.9^a^	40.1^a^	1.82	<0.01
48 h	34.8	41.0	46.9	2.53	0.22
ADF					
24 h	23.8^b^	32.7^a^	37.8^a^	2.14	<0.01
48 h	26.5	37.2	43.5	3.16	0.06
pH					
24 h	6.46	6.49	6.53	0.020	0.10
48 h	6.54	6.55	6.58	0.020	0.76
GP_48h_ (mL/g)	141.6	147.3	158.5	3.67	0.18
A (mL)	145.9^b^	157.3^ab^	163.2^a^	4.57	0.05
B	1.47	1.28	1.21	0.075	0.37
C (h)	6.34	5.88	6.42	0.299	0.75

a,b *Values denoted by different superscript letters in the same row indicate significant differences (p < 0.05), whereas those denoted by the same letters or no letters indicate no significant differences (p > 0.05). SEM, standard error of the mean*.

### Feed Intake and Nutrient Digestibilities

The DMI values of the SP-supplemented group and CON group were 25.98 and 26.66 kg/ (cow·day), respectively (Table 4). The nutrient apparent total tract digestibilities of ADF were decreased when cows were supplemented with SPs (*p* > 0.05), whereas nutrient digestibilities of DM, CP, and NDF were increased (*p* > 0.05) (Table 4).

**Table 4:** Effects of the dietary supplementation of soybean peptides (SPs) on feed intake and apparent total tract digestibility in lactating dairy cows.

**Item^**a**^**	**Treatment** ^**b**^		**SEM**	***P***
	**CON**	**SPs**		
DMI, kg/day	26.0	26.7	0.15	–
DM, %	66.2	67.1	3.14	0.72
CP, %	63.0	64.6	3.23	0.67
NDF, %	52.5	54.1	3.33	0.32
ADF, %	52.1	49.9	3.42	0.61

a *DMI, dry Matter Intake; DM, dry Matter; CP, Crude Protein; NDF, Neutral Detergent Fiber; ADF, Acid Detergent Fiber*.

b *CON, the Control Group With no Soybean Peptide Supplementation; SPs, the Group With 50 g/Head/day of Soybean Peptide Supplementation*.

### Milk Yield and Components

The results of milk yield and components were shown in Table 5. Milk yield in the SP-supplemented group was 35.5 kg/day, which was significantly higher than 34.6 kg/day in the CON group (*p* < 0.05), but no effects on the 3.5% FCM and ECM were found between the two groups (*p* > 0.05). Percentages of milk components, including protein and lactose, were not affected by SPs supplementation (*p* > 0.05), but milk fat percentage was decreased (*p* < 0.05). Milk protein yield (*p* = 0.01) and milk lactose yield (0.05 < *p* < 0.10) were increased when cows were supplemented with SPs. Except for milk protein and lactose percentage, supplemented time effects were detected for all variables (*p* < 0.01). An interaction between treatment and time was only observed for the milk protein percentage (*p* < 0.01).

**Table 5:** Effects of the dietary supplementation of soybean peptides (SPs) on milk yield and milk components in lactating dairy cows.

**Item^**a**^**	**Treatment** ^**b**^		**SEM**	***P***		
	**CON**	**SPs**		**Treatment**	**Time**	**Treatment × Time**
Milk yield, kg/day	34.6	35.5	0.12	0.04	<0.01	0.55
3.5% FCM, kg/day	39.5	41.0	0.34	0.81	<0.01	0.45
ECM, kg/day	39.5	40.1	0.31	0.41	<0.01	0.42
Protein, %	3.26	3.28	0.014	0.52	0.20	<0.01
Protein, kg/day	1.12	1.17	0.008	0.01	<0.01	0.14
Fat, %	4.63	4.47	0.040	0.03	<0.01	0.62
Fat, kg/day	1.58	1.59	0.017	0.93	<0.01	0.67
Lactose, %	5.03	5.04	0.013	0.83	0.03	0.63
Lactose, kg/day	1.75	1.79	0.011	0.07	<0.01	0.87

a *3.5% FCM = Milk Yield (kg) × 0.4324 + Milk fat (kg) × 16.218. ECM = milk yield (kg) × 0.3246 + milk fat (kg) × 12.86 + milk protein (kg) × 7.04*.

b *CON, the Control Group With no Soybean Peptide Supplementation; SPs, the Group With 50 g/Head/day of Soybean Peptide Supplementation*.

### Rumen Fermentation Parameters

There was no significant difference in the rumen pH and NH_3_-N concentration between the two groups (*p* > 0.05), whereas MCP concentration tended to increase in the SP-supplemented group (0.05 < *p* < 0.10) (Table 6). Furthermore, the ruminal concentrations of TVFA, acetate, propionate, butyrate, and valerate were significantly higher in the SP-supplemented group than in the CON group (*p* < 0.05). However, SPs supplementation had no effect on molar proportions of the four individual VFAs and the acetate/propionate ratio (*p* > 0.05).

**Table 6:** Effects of the dietary supplementation of soybean peptides (SPs) on the rumen fluid fermentation profile of lactating dairy cows on the last day of the experiment.

**Parameter^**a**^**	**Treatment** ^**b**^		**SEM**	***P***
	**CON**	**SPs**		
pH	6.33	6.51	0.117	0.48
NH_3_-N (mg/dL)	14.8	12.3	0.99	0.23
MCP (mg/mL)	1.64	1.67	0.050	0.09
TVFA (mmol/ml)	95.2	113	3.57	0.01
Acetate (mmol/mL)	59.2	70.0	2.12	0.01
Propionate (mmol/mL)	23.8	28.1	1.05	0.04
Butyrate (mmol/ mL)	8.92	10.8	0.398	0.02
Valerate (mmol/ mL)	1.46	1.70	0.053	0.02
Acetate, %	62.2	62.3	0.36	0.91
Propionate, %	24.9	24.9	0.41	0.96
Butyrate, %	9.39	9.52	0.150	0.67
Valerate, %	1.53	1.52	0.024	0.87
Acetate: Propionate ratio	2.53	2.51	0.053	0.88

a *NH_3_-N, Ammonia Nitrogen; MCP, Microbial Crude Protein; TVFA, Total Volatile Fatty Acid*.

b *CON, the Control Group With no Soybean Peptide Supplementation; SPs, the Group With 50 g/Head/day of Soybean Peptide Supplementation*.

### Blood Parameters

Soybean peptide supplementation significantly increased the concentrations of blood parameters, including energy metabolism, oxidative stress indicators, and immunity parameters (Table 7). Soybean peptide supplementation significantly increased the concentrations of blood parameters, including energy metabolism, oxidative stress indicators, and immunity parameters. As for energy metabolism, SPs supplementation tended to increase the TAA concentration (0.05 < *p* < 0.10) and significantly increased the concentration of Glu and BHBA (*p* = 0.05). The oxidative stress indicators SOD (*p* < 0.01) and IgG (*p* < 0.05) were significantly higher in the SP-supplemented group than in the CON group.

**Table 7:** Effects of the dietary supplementation of soybean peptides (SPs) on the blood parameters of lactating dairy cows on the last day of the experiment.

**Parameter^**a**^**	**Treatment** ^**b**^		**SEM**	***P***
	**CON**	**SPs**		
**Energy metabolism**				
Ins, μIU/ml	23.8	27.3	1.35	0.20
GLU, mmol/L	3.65	3.91	0.066	0.05
TG, mmol/L	0.180	0.181	0.0052	0.93
TC, mmol/L	6.20	6.98	0.330	0.25
BHBA, mmol/L	0.264	0.288	0.0063	0.05
NEFA, μmol/L	32.1	32.2	0.69	0.96
TAA, μmol/mL	6.09	7.92	0.506	0.07
**Oxidative stress**				
SOD, U/mL	137	159	3.8	<0.01
GSH-Px, μmol/L	13.3	16.7	1.19	0.16
CAT, U/mL	11.9	13.5	0.78	0.32
**Immunity parameters**				
IgA, μg/mL	430	397	10.2	0.10
IgG, μg/mL	11.4	12.9	0.35	0.03
IgM, μg/mL	3.72	3.54	0.072	0.22

a *Ins, Insulin; GLU, Glucose; TG, Triglyceride; TC, Total Cholesterol; BHBA, Beta-Hydroxybutyric Acid; NEFA, non-Esterified Fatty Acid; TAA, Total Amino Acid; SOD, Superoxide Dismutase; GSH-Px, Glutathione Peroxidase; CAT, Catalase; IgA, Immunoglobulin A; IgG, Immunoglobulin G; IgM, Immunoglobulin M*.

b *CON, the Control Group With no Soybean Peptide Supplementation; SPs, the Group With 50 g/Head/day of Soybean Peptide Supplementation*.

### Ruminal Bacterial Communities

#### Alpha-Diversity of Rumen Bacterial Communities

**Figure 1:**
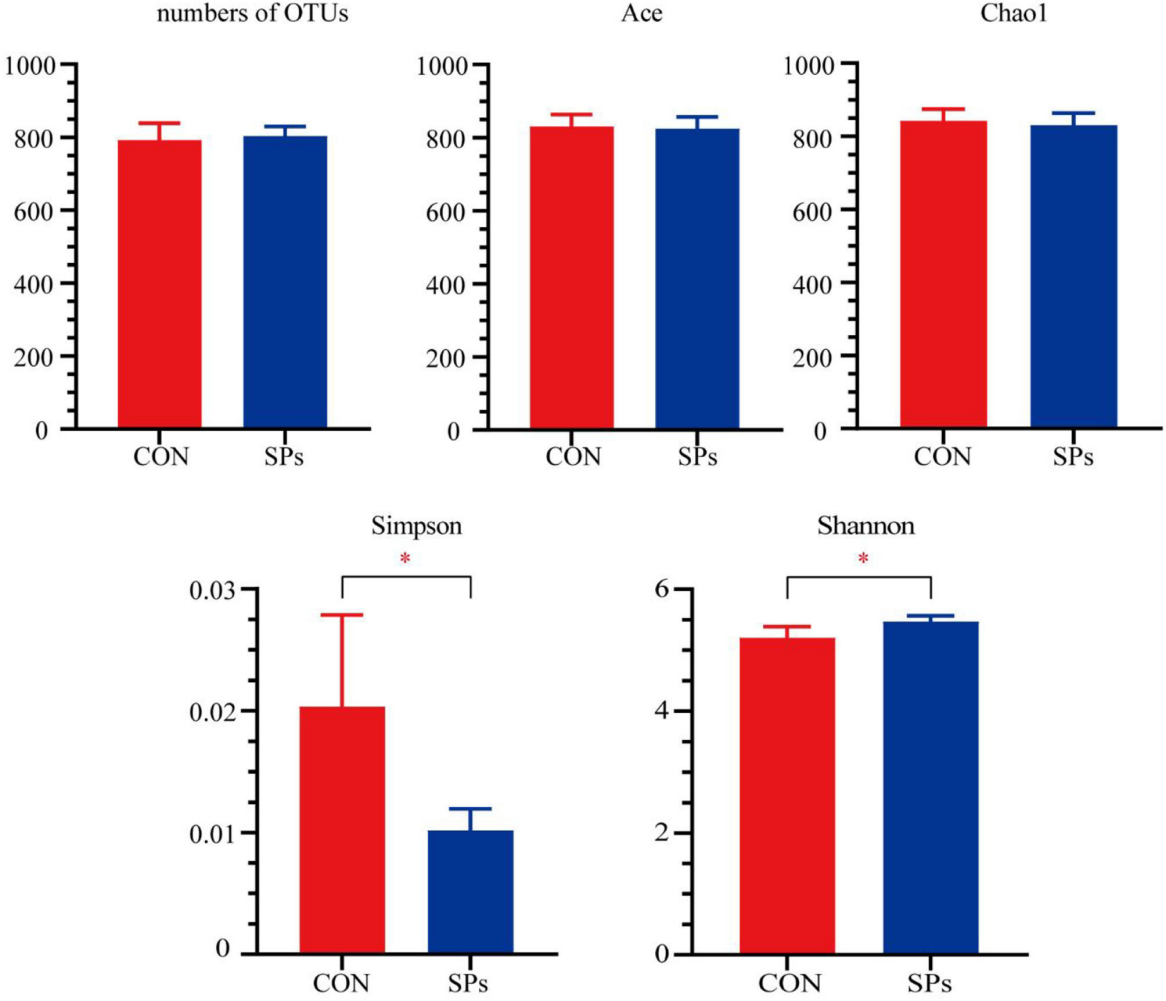
Effects of the dietary supplementation of soybean peptides (SPs) on the alpha-diversity of rumen microbial communities. ^*^, significant differences (*p* < 0.05). CON, the control group with no soybean peptide supplementation; SPs, the group with 50 g/head/day of soybean peptide supplementation.

A total of 277,160 qualified sequences assigned to 860 OTUs were generated from 10 rumen fluid samples after data filtering. The coverage rate of the bacterial communities in each sample was >99.00%, implying a sufficient sequence depth to accurately describe bacterial composition. The alpha-diversity indices are plotted in Figure 1. The number of OTUs and values of Chao1 and Ace were not significantly affected by SPs supplementation (*p* > 0.05), whereas the Simpson (*p* < 0.05) and Shannon (*p* = 0.01) indices showed significant differences among the two groups.

#### Beta-Diversity of Rumen Bacterial Communities

**Figure 2:**
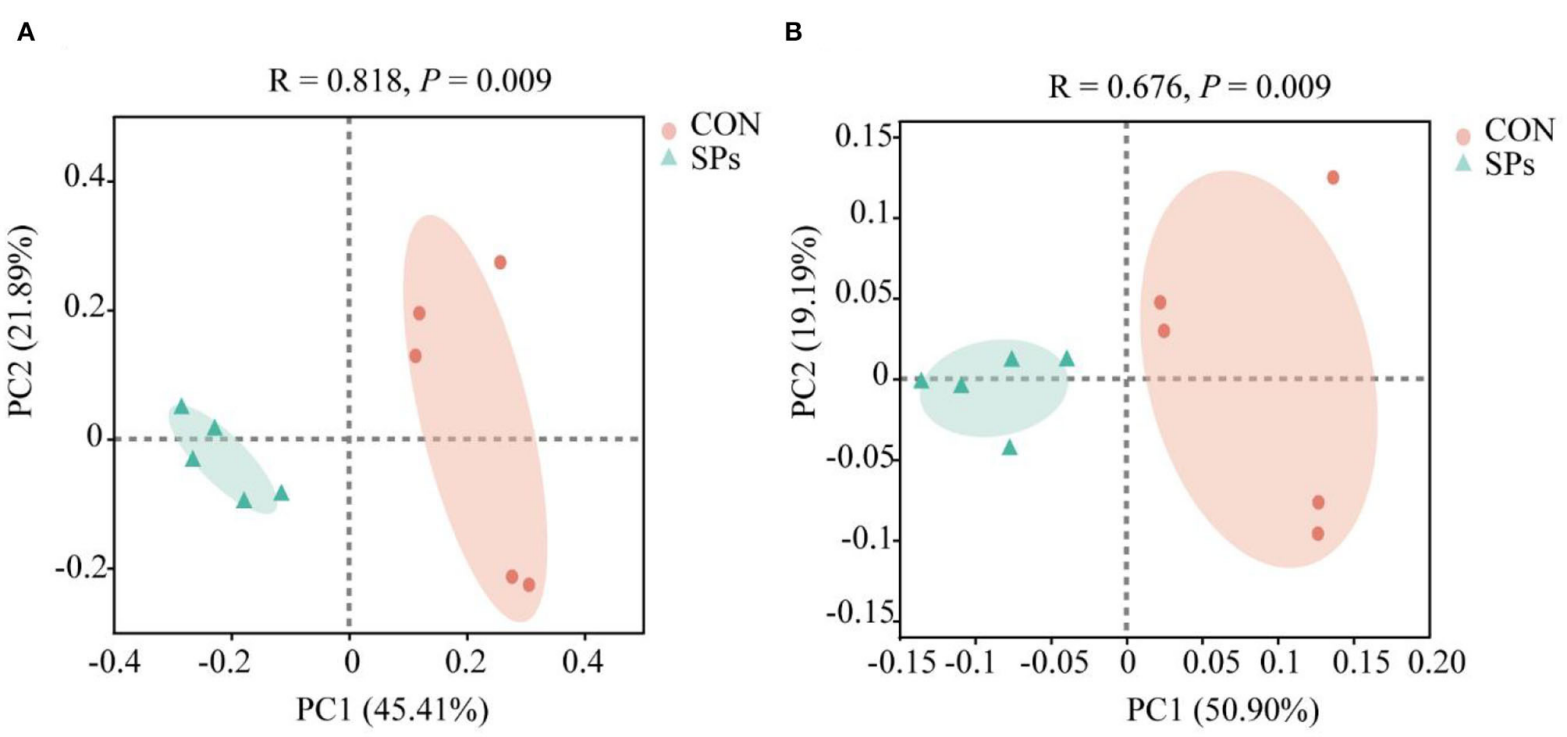
Effects of the dietary supplementation of soybean peptides (SPs) on beta-diversity according to OTU levels. **(A)** PCoA of ruminal bacteria based on the Bray–Curtis distance. **(B)** PCOA of ruminal bacteria based on Binary Jaccard distance. CON, the control group with no soybean peptide supplementation; SPs, the group with 50 g/head/day of soybean peptide supplementation.

**Figure 3:**
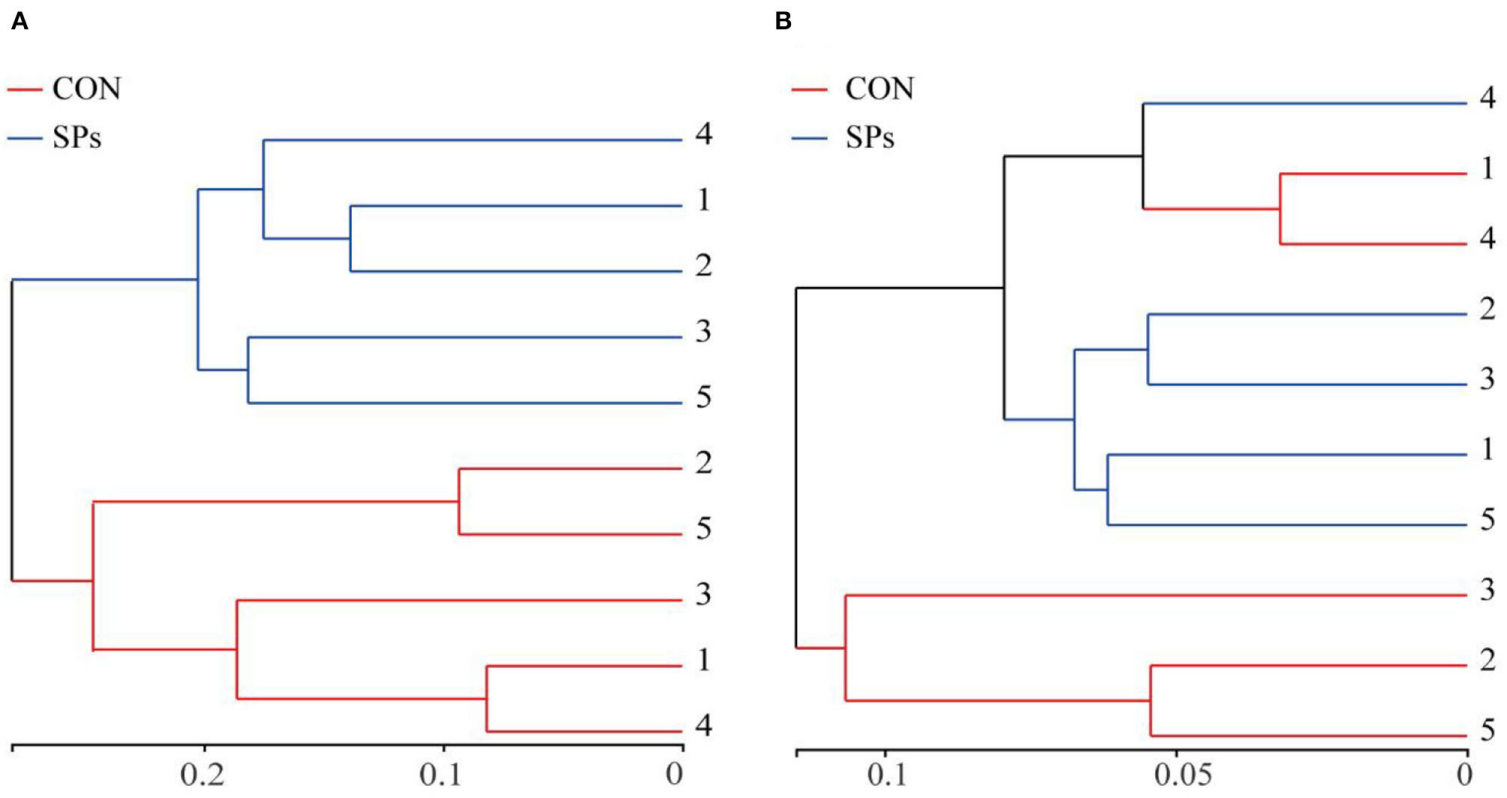
Hierarchical clustering according to OTU levels. **(A)** Hierarchical clustering of ruminal bacteria based on Bray – Curtis distance. **(B)** Hierarchical clustering of ruminal bacteria based on Binary Jaccard distance. CON, the control group with no soybean peptide supplementation; SPs, the group with 50 g/head/day of soybean peptide supplementation.

The results of the PCoA with Bray–Curtis distance (*R* = 818, *p* = 0.009) and binary Jaccard distance (*R* = 676, *p* = 0.009) indicated that the bacterial communities of the two groups were visually separated from each other at the OTU level (Figure 2). The hierarchical clustering analysis based on the OTU levels of the two groups is shown in Figure 3. The ruminal bacteria of the SP-supplemented and CON groups formed two separate hierarchical clusters according to OUT level based on the Bray–Curtis distance.

#### Composition of Ruminal Bacteria

**Figure 4:**
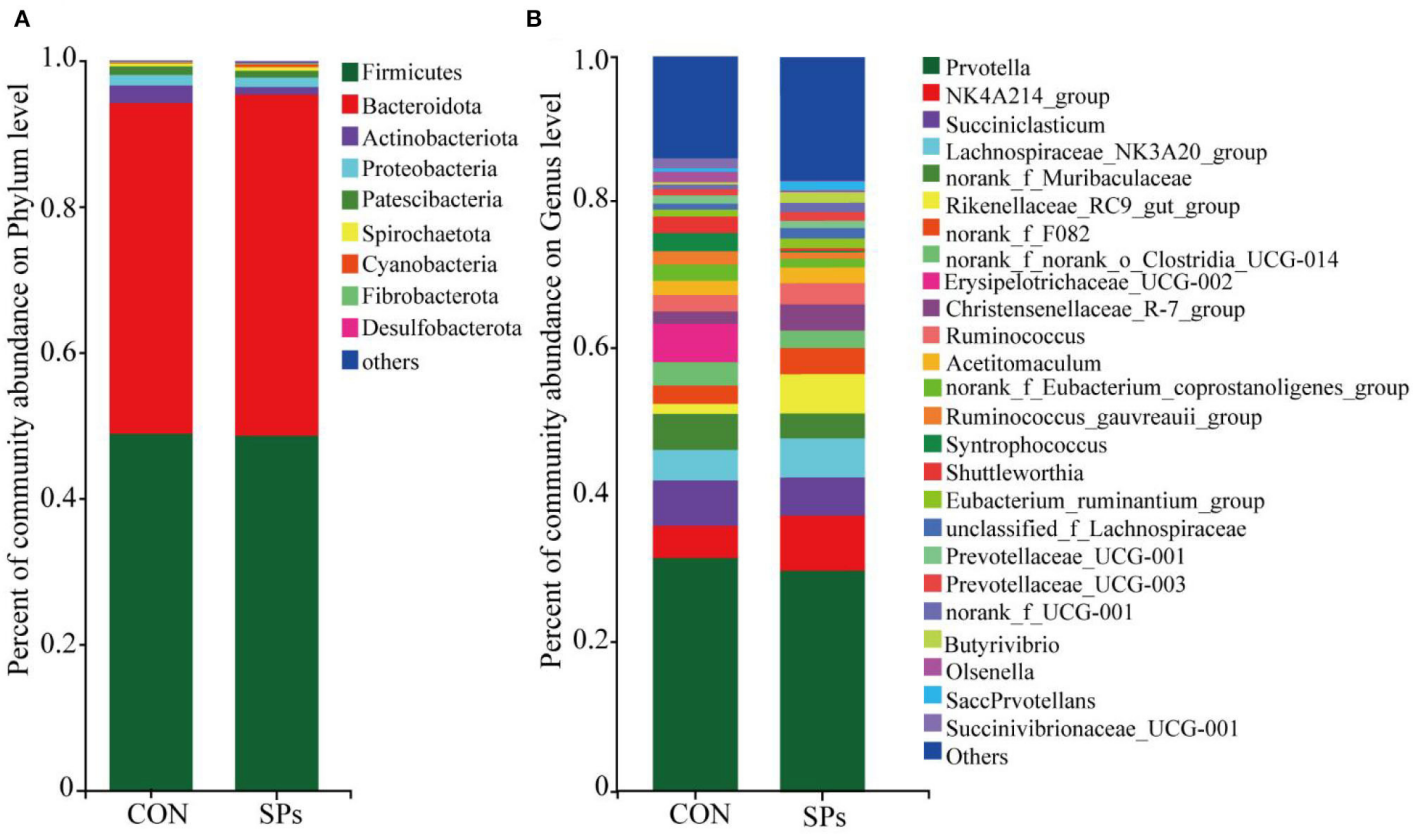
Effects of the dietary supplementation of soybean peptides (SPs) on the composition of **(A)** phyla and **(B)** genera of bacterial communities in CON and SPs groups. CON, the control group with no soybean peptide supplementation; SPs, the group with 50 g/head/day of soybean peptide supplementation.

The relative abundances of rumen bacteria are shown in Figure 4. The dominant bacterial phylum (relative abundance > 1%) was *Firmicutes* (48.77% ± 11.72%), followed by *Bacteroidetes* (46.04% ± 11.60%), *Actinobacteriota* (1.72% ± 1.49%), *Proteobacteria* (1.37% ± 1.58%), and *Patescibacteria* (1.05% ± 0.44%). The top 25 genera with relative abundances >1% are also shown in Figure 4.

**Figure 5:**
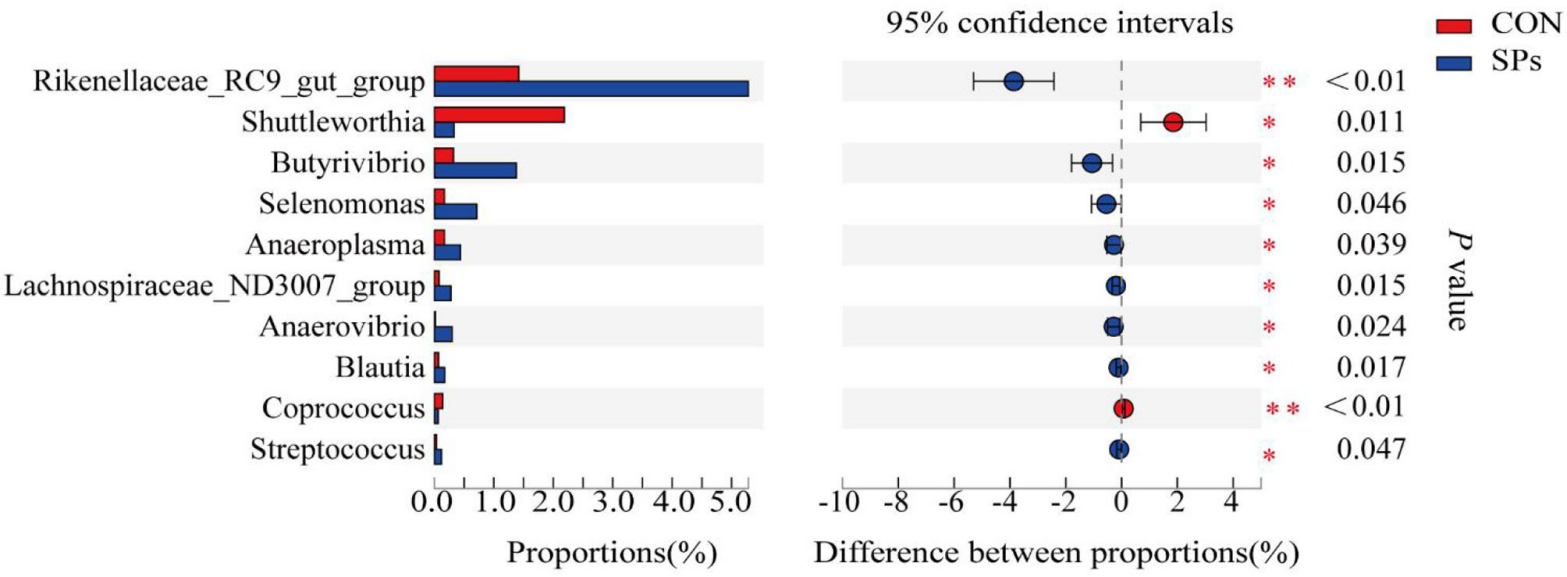
Effects of the dietary supplementation of soybean peptides (SPs) on genera relative abundance in CON and SPs groups. *, significant differences (*p* < 0.05); **, highly significant differences (*p* < 0.01). CON, the control group with no soybean peptide supplementation; SPs, the group with 50 g/head/day of soybean peptide supplementation.

The top 10 clearly classified genera in the two groups are shown in Figure 5. All these genera belonged to *Firmicutes*, except for *Rikenellaceae_RC9_gut_group*. Compared with the CON group, the SP-supplemented group had significantly increased relative abundance of *Rikenellaceae_RC9_gut_group* (*p* < 0.01), *Butyrivibrio, Selenomonas, Anaeroplasma, Lachnospiraceae_ND3007_group, Anerovibrio, Blautia*, and *Streptococcus* (*p* < 0.05). In contrast, the relative abundance of *Shuttleworthia* (*p* < 0.05) and *Coprococcus* (*p* < 0.01) was decreased with SP supplementation.

#### PICRUSt2 Analysis

**Figure 6:**
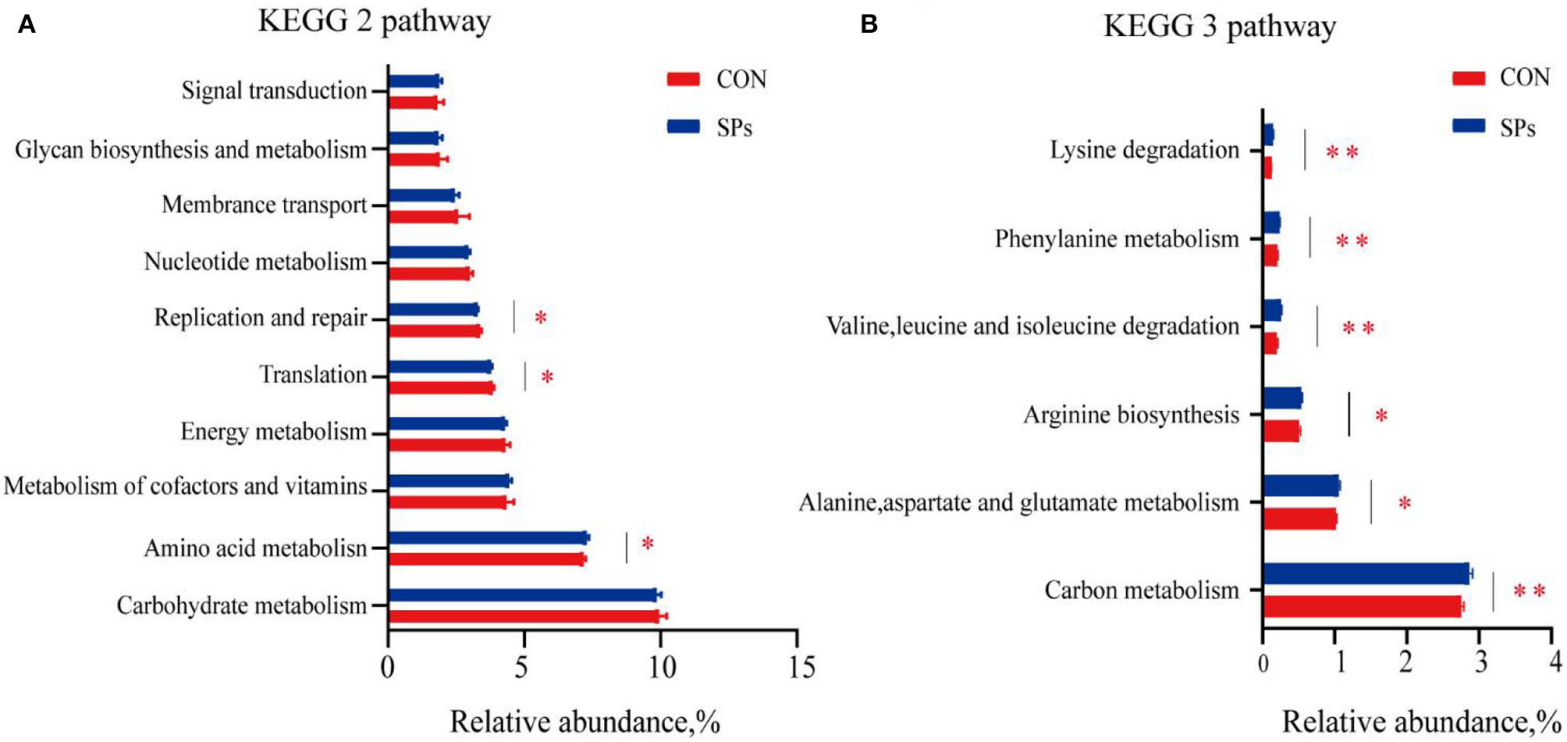
Predicted bacterial functions using PICRUSt analysis in CON and SP groups. **(A)** KEGG level 2 pathways. **(B)** KEGG level 3 pathways of “Global and overview maps” and “Amino acid metabolism” in the two groups. *, significant differences (*p* < 0.05); **, highly significant differences (*p* < 0.01). CON, the control group with no soybean peptide supplementation; SPs, the group with 50 g/head/day of soybean peptide supplementation.

The predicted functions of rumen bacteria were analyzed using PICRUSt2. A number of ten most important functions were detected for ruminal bacteria (Figure 6). These functions were “Carbohydrate metabolism,” “Amino acid metabolism,” “Metabolism of cofactors and vitamins,” “Energy metabolism,” “Translation,” “Replication and repair,” “Nucleotide metabolism,” “Membrane transport,” “Glycan biosynthesis and metabolism,” and “Signal transduction.” For the SP-supplemented group, the “Amino acid metabolism” predicted the function of the KEGG level 2 pathway was increased, whereas “Translation” and “Replication and repair” were decreased compared with those in the CON group (*p* < 0.05). “Alanine, aspartate, and glutamate metabolism” (*p* < 0.05), “Arginine biosynthesis” (*p* < 0.05), “Valine, leucine, and isoleucine degradation” (*p* < 0.01), “Phenylalanine metabolism” (*p* < 0.01), and “Lysine degradation” (*p* < 0.01) and the KEGG level 3 pathway of “Amino acid metabolism” were significantly upregulated in the SP-supplemented group. “Carbon metabolism” of the KEGG level 3 pathway, that is the subway of “Global and overview maps,” was significantly increased in the SP-supplemented group compared with that in the CON group (*p* < 0.01).

## Discussion

### *In vitro* Experiment

The *in vitro* experiment aimed to determine an effective addition level of SPs for further application in animal feeding. The *in vitro* degradability of nutrients has been extensively used as an indicator of their degradability *in vivo* (39). Gas production *in vitro* is an index of the fermentation degree of feedstuffs and activity of rumen microbes (40). Our results showed that the group supplemented with 1.92 g/kg DM of SPs showed the highest degradability of DM, NDF, ADF, and CP of TMR at 24 and 48 h. The results of DM degradability were anticipated because a previous *in vitro* experiment documented that DM degradability was increased with the addition of SPs ranging from 0 to 0.75% (DM basis) after 24 and 48 h of incubation (8). The total tract digestion of NDF and ADF was also increased with ruminal small peptides infusion at the rates of 100, 200, and 300 g/d, and it mainly contributed to the improved rumen fermentation ability (41). Nutrients are degraded by microorganisms in the rumen; thus, the elevated degradability may be related to the enhanced microbial activity. Peptides can be degraded and deaminated to ammonia, and SPs can facilitate the growth of the rumen microbe, in particular, of cellulolytic bacteria that use ammonia for microbial protein synthesis (41); thus, 1.92 g/kg DM of SPs may provide a richer ruminal pool of ammonia-N and resulted in higher NDF and ADF degradability at two time points in our study. However, these observations were different from those of Wang et al. (8), who showed that NDF and ADF degradabilities decreased after 48-h incubation with increasing SPs inclusion up to 0.75% (DM basis). The inconsistent results may be attributed to the differences in the basal diet and fiber concentration (8), addition level of peptides (8, 42), composition of peptides (43), and dietary concentrate to forage ratio (42). Rumen pH is an important index for measuring the rumen fermentation environment. Ruminal pH was lower with soybean peptides (8) and tri-peptides addition (44), whereas in our study, rumen pH remained constant at two measuring time points. The various findings may result from the differences in rumen fluid donors, substrate composition, and peptide amino acid composition. The total and asymptotic gas production at high levels of SPs (1.92 g/kg DM of SPs) was the highest, and it could positively indicate a corresponding high DM degradability. In line with the current *in vitro* results, 1.92 g/kg DM of SPs generally improved the degradability of nutrients without changing the rumen fermentation environment; therefore, the *in vivo* experiment was conducted.

### *In vivo* Experiment

In the *in vitro* experiment, 1.92 g/kg DM of SPs supplementation showed the greatest digestibility and gas production. The amounts of additional SPs were calculated according to 26 kg DM in the TMR, and 1.92 g/kg DM of SPs was equivalent to 50 g/head/day SPs supplementation in animal feeding experiment. Thus, the *in vivo* experiment was carried out to investigate the effects of 0 and 50 g/head/day SPs supplementation on lactating dairy cows. Our experiment was conducted with large-scale herd in two separate barns, and the influence of barns was an uncontrollable factor in the theoretical study. Nevertheless, the herd protocol including feeding time and milking process was the same between the two barns, which could mitigate the effects of barns.

Soybean peptide supplementation had no effects on the DM and NDF digestibility, and the DMI of cows with SPs supplementation was higher compared to that of the control diet. In contrast, ruminal infusion of soybean small peptides at 100, 200, and 300 g/d was reported to increase the ATTD of DM and NDF in cattle (41). The discrepancy in nutrient digestibility between these results may be due to the addition level of soybean peptides and the NDF and ADF concentrations of the basal diets. In dairy cows, the positive response of DMI was consistent with higher NDF digestibility (45). In this study, the chemical composition of the basal diet and NDF digestibility were consistent between the two groups. Cows fed SPs had higher yields of milk and milk components; thus, we speculated that the DMI in the SPs group was increased to achieve the nutrient and energy requirements of increased milk production. In addition, the improved DMI provided more substrate to rumen microorganisms and produced more VFAs. These findings highlight the potential of SPs to stimulate nutrient intake and maintain a better nutrient utilization status of the body. However, we should be aware that the DMI in our study was averaged for the whole barn and was not provided for individual cows; thus, we did not perform statistical analysis of DMI. However, there was a difference of almost 0.7 kg between the two groups numerically. Meanwhile, to reduce the stress to cows caused by sampling, fecal samples were collected for 3 days, which is a short time for digestibility calculation and may not reflect the effects of SPs on digestibility accurately.

Supplemental SPs decreased the milk fat percentage from 4.63 to 4.57%, but the milk fat yield was not affected. The ruminal concentrations of acetate (59.19 vs. 69.95 mmol/ml) and butyrate (8.92 vs. 10.75 mmol/ml) for the CON and SP-supplemented groups may reflect the ability of mammary epithelial cells to synthesize fatty acids *de novo* (46), thus promoting milk fat yield. We hypothesized that the unexpected lower milk fat percentage in the SP-supplemented cows was due to the improved milk yield.

Milk protein and milk lactose percentages were not influenced by SPs supplementation; however, the yields of milk protein and lactose were significantly higher in the SP-supplemented group than in the CON group. Milk protein is closely associated with dietary CP levels and specific essential amino acid (EAA) supplementation (47). Previous research showed that ruminal infusion of soybean small peptides increased the flux of each of 17 AAs, TAAs, and essential AAs (EAAs) to the small intestine (41). We supposed that SPs may be broken down to amino acids in the rumen and the small intestine, but when the basal diet with 16.7% CP met the nutritional requirement of dairy cows, the SPs had no beneficial effects on protein synthesis. In our study, the lower NH_3_-N and higher MCP in the rumen of SP-supplemented cows may lead to the elevated concentration of metabolizable protein flowing to the small intestine. MCP combined with undegradable SPs provides more absorbed available AAs in the blood, which are removed by the mammary cells from the vascular supplies to synthesize milk protein. As far as our experiment is concerned, the ruminal degradability of soybean peptides needs further investigation. Milk lactose synthesis is influenced by udder health (48), metabolism, and energy balance in dairy cows (49). Propionate accounts for half of the hepatic glucose output, which in turn accounts for two-thirds of the glucose required for milk lactose synthesis (50, 51). Therefore, the higher milk lactose yield in the SP-supplemented group was derived from its higher ruminal propionate concentration (28.08 mmol/L) when compared to the CON group. As an important osmotic component of milk, lactose drives the secretion of water into the mammary glands from the bloodstream (49). Thus, the yield of milk lactose was the main reason for the improvement in the milk yield.

Glucogenic AAs include most AAs and can be converted into glucose in the liver. TAA and plasma glucose were higher in SP-supplemented cows, which exhibited better gluconeogenesis in the liver induced by AAs. Plasma BHBA is an incomplete oxidation product of NEFA in the liver. Blood BHBA concentration is detected to determine hyperketonemia in early lactation, and most researchers apply 1.2 mmol/L of BHBA as the threshold of hyperketonemia (52). The BHBA concentration in the two groups of our *in vivo* experiment was within the acceptable limits (<1.2 mmol/L), although the SP-supplemented group tended to present higher BHBA concentration than the CON group. Butyrate is the precursor of BHBA, and an elevated BHBA concentration may have been derived from the higher butyrate concentration in the rumen of SP-supplemented cows than CON cows (46). The concentration of antioxidant substances in the SP-supplemented group was higher than that in the CON group. GSH-Px, SOD, and CAT are considered oxidative stress markers and are crucial antioxidant enzymes in biological systems (53, 54). The antioxidant capacity of peptides depends on their AAs profile, and methionine, histidine, lysine, and tryptophan are considered to be antioxidative AAs (55). The underlying mechanism is that more active R bases of amino acids in soybean protein are exposed during hydrolysis (55). Plasma IgA concentration tended to decrease, and IgG concentrations increased when SPs were fed to cows. IgG is the predominant antibody in blood and plays an essential role in humoral immunity (56). SPs have shown immunomodulatory activity *in vitro* (5). Furthermore, SPs reduce the impact of coccidia challenge by downregulating the expression of plasma IgA in broiler chickens (16). In this study, the fluctuation of plasma IgA within the normal range (21, 26) along with the elevated IgG concentration could reflect the healthier status of SP-supplemented cows.

Rumen harbors a complex community of microorganisms, and the VFA concentration in the rumen depends on the fermentation rate of diet components by the microbial community and the respective energy provision rate to dairy cows. TVFAs and individual VFAs were higher in SP-supplemented cows than in CON cows. Increased concentration of TVFAs agrees with the results of previous reports, in which SPs were either added *in vitro* or infused ruminally (8, 41). The acetate/propionate ratio did not change in this study, reflecting a stable fermentation pattern, but it was inconsistent with that observed in previous studies (8). The discrepancy in the effects of SPs on the rumen fermentation profile might be due to the supplementation method, experimental animal, basal diet composition, and supplementation level.

We also studied the effects of SPs supplementation on the rumen bacterial community of dairy cows. As mentioned previously, to reduce the stress of sampling and influence of herd protocol in the farm, we only selected five cows from each group for rumen fluid collection and analysis of microbial communities. Although the dairy cows we selected were under healthy physiological conditions and had similar production performance, the sample size should be expanded in the future to reduce the variability. From the results, significant differences in the structure of microorganisms emerged between the two groups, suggesting that SPs can modulate the rumen bacterial community. In addition, *Firmicutes* and *Bacteroidetes* were the dominant microorganisms at the phylum level, which is consistent with ruminal bacteria studies (57, 58). The results of alpha-diversity implied that bacterial richness was improved by SPs supplementation and beta-diversity showed that SPs play distinct roles in regulating the bacterial community. The relative abundance of *Butyrivibrio* was increased in the SP-supplemented group, similar to that previously reported for the relative abundance of *Butyrivibrio fibrisolvens* using real-time PCR (8). *Bacteria* within the *Butyrivibrio* genus are cellulolytic bacteria, and their fermentative product in the rumen is mainly butyrate. SPs supplementation has been shown to improve the growth of acetate-producing bacteria such as *Rikenellaceae_RC9_gut_group* and *Lachnospiraceae_ND3007_group* (59, 60). Bacteria associated with cellulose degradation were also stimulated by SPs supplementation. The *Rikenellaceae_RC9_gut_group* was found to be related to structural carbohydrate fermentation (59), and *Anaeroplasma* was related to crude fiber digestibility in pigs (60). The relative abundances of these two fibrolytic bacteria were higher in the SP-supplemented group than in the CON group. Therefore, SPs supplementation may improve cellulose degradation through microbial regulation. The explicit function of *Shuttleworthia* is unclear. Hao et al. (61) found that *Shuttleworthia* was highly positively correlated with MCP and short-chain VFA production in postweaning calves. However, *Shuttleworthia* is a pathogen in the gut of rats, and its level decreases when rats are fed prebiotic isomaltulose (62). Ruminal microbiota are complex and interactive, and the role of *Shuttleworthia* in ruminants and monogastric animals needs to be explored in the future. We speculated that the lower relative abundance of *Shuttleworthia* in the SP-supplemented group may indicate a healthier body condition, which is supported by increased blood immunity parameters. The researchers found that nitrate supplementation enhanced ammonia incorporation into *Selenomonas* microbial protein in dairy cows (63, 64), so the higher relative abundance of *Selenomonas* may reflect the higher concentration of ammonia-N derived from degradative SPs that were incorporated in the *Selenomonas*.

As expected, PICRUSt revealed that the predicted functions of bacteria in the AA metabolic pathways are different among the two groups. SPs are a mixture of AAs, and the concentration of individual AAs is different. Valine, leucine, and isoleucine are branched-chain amino acids (BCAAs), which are vital for milk protein synthesis and glycolipid metabolism (65). SPs supplementation significantly increased the predicted functions of valine, leucine, and isoleucine degradation, which may explain the enhanced microbial CP synthesis and the energy regulation of the body in SP-supplemented cows.

## Conclusions

Soybean peptides supplemented at the level of 1.92 g/kg DM have the potential to increase DM, CP, NDF, and ADF degradabilities after 24 and 48 h of fermentation *in vitro*. In the *in vivo* study, at 50 g/head/day supplementation level, SPs tended to increase DMI, which resulted in improved milk production. The elevated milk lactose yield, originating from increased rumen propionate and blood glucose concentration, also contributed to the improvement of milk production in SP-supplemented cows. About 50 g of SPs per day also improved the antioxidant capacity and immunity of dairy cows. We observed that dietary SPs supplementation improved rumen bacterial richness, thus resulting in a different structure of the community. The relative abundances of cellulolytic bacteria, such as *Rikenellaceae_RC9_gut_group* and *Butyrivibrio*, were increased in the SP-supplemented group, and *Selenomonas* was increased because of the strong capacity of peptides, AA utilization, and VFA productivity. The predicted AA metabolic pathway functions of bacteria were influenced by SPs supplementation, but the relationship between the amino acid composition of SPs and the ruminal amino acid degradation needs further investigation. In addition, the *in vivo* experiment was conducted with a large-scale herd, which was limited by a small number of sampling cows. Therefore, more frequent sampling is required to further determine the role of added SPs on dairy cows.

## Data Availability Statement

The datasets presented in this study can be found in online repositories. The names of the repository/repositories and accession number(s) can be found at: https://www.ncbi.nlm.nih.gov/, BioProject accession ID: 779612, RUN: SRR 18218289 – SRR 18218298.

## Ethics Statement

All experimental procedures were approved by the Institutional Review Board of the China Agricultural University (Protocol number: AW81102202-1-3 and 28.4.2017 of approval). Animal welfare and handling procedures were followed by China Agricultural University experimental guidelines.

## Author Contributions

Conceptualization: TX, FK, and ZC. Methodology and investigation: TX and FK. Data curation and writing – original draft preparation: TX. Formal analysis: FK and WW. Writing, reviewing, and editing: WW, YW, and SL. Resources: WW and HY. Project administration: SL. Supervision: WW, HY, and SL. All authors have read and agreed to the published version of the manuscript.

## Funding

This research was funded by the National Natural Science Foundation of China, Grant Number 32130100; China Agriculture Research System of Ministry of Finance and Ministry of Agriculture and Rural Affairs (CARS 36), the 2115 Talent Development Program of China Agricultural University.

## Conflict of Interest

The authors declare that the research was conducted in the absence of any commercial or financial relationships that could be construed as a potential conflict of interest.

## Publisher's Note

All claims expressed in this article are solely those of the authors and do not necessarily represent those of their affiliated organizations, or those of the publisher, the editors and the reviewers. Any product that may be evaluated in this article, or claim that may be made by its manufacturer, is not guaranteed or endorsed by the publisher.

## References

[1] Sun P Li DF Dong B Qiao SY Ma X. Effects of soybean glycinin on performance and immune function in early weaned pigs. Arch Anim Nutr. (2008) 62:313–21. 10.1080/17450390802066419 18763625

[2] Lalles JP Dreau D Femenia F Parodi AL Toullec R. Feeding heated soyabean flour increases the density of B and T lymphocytes in the small intestine of calves. Vet Immunol Immunop. (1996) 52:105–15. 10.1016/0165-2427(95)05534-7 8807780

[3] Wright DE. Metabolism of peptides by rumen microorganisms. Appl Microbiol. (1967) 15:547–50. 10.1128/am.15.3.547-550.1967 6035045 PMC546966

[4] Newey H Smyth DH. Intracellular hydrolysis of dipeptides during intestinal absorption. J Physiol-London. (1960) 152:367–80. 10.1113/jphysiol.1960.sp006493 14426799 PMC1363321

[5] Wen LR Jiang YM Zhou XS Bi HM Yang B. Structure identification of soybean peptides and their immunomodulatory activity. Food Chem. (2021) 359:129970. 10.1016/j.foodchem.2021.129970 34015561

[6] Zhao YB Zhang TL Zhang YM Bai C Ao CJ. Effects of enzymatic hydrolysate of cottonseed protein on rumen fermentation and microflora of dairy cows *in vitro*. Chin J Anim Nutr. (2021) 33:3297–308. 10.3969/j.issn.1006?267x.2021.06.032

[7] IlSup K WoongSuk Y CheorlHo K. Beneficial effects of soybean-derived bioactive peptides. Int J Mol Sci. (2021) 22:8570. 10.3390/ijms22168570 34445273 PMC8395274

[8] Wang L Liu Y Liu S Sun K Wang X Zhang G. Effect of soybean peptides on *in vitro* ruminal fermentation and microbial population. J Northeast Agri Univ. (2017) 24:40–52.

[9] Zhao WZ Xue SY Yu ZP Ding L Li JR Liu JB. Novel ACE inhibitors derived from soybean proteins using in silico and *in vitro* studies. J Food Biochem. (2019) 43:e12975. 10.1111/jfbc.12975 31489673

[10] Li WH Li H Zhang YX He LJ Zhang C Liu XQ. Different effects of soybean protein and its derived peptides on the growth and metabolism of bifidobacterium animalis subsp. animalis JCM 1190. Food Funct. (2021) 12:5731–5744. 10.1039/D1FO00480H 34132282

[11] Zhang C Xia SQ Zhang YX Zhu SY Li H Liu XQ. Identification of soybean peptides and their effect on the growth and metabolism of Limosilactobacillus reuteri LR08. Food Chem. (2022) 369:130923. 10.1016/j.foodchem.2021.130923 34455331

[12] Yi GF Din JU Zhao F Liu XQ. Effect of soybean peptides against hydrogen peroxide induced oxidative stress in HepG2 cells via Nrf2 signaling. Food Funct. (2020) 11:2725–37. 10.1039/C9FO01466G 32167099

[13] Rayaprolu SJ Hettiarachchy NS Horax R Phillips GK Mahendran M Chen PY. Soybean peptide fractions inhibit human blood, breast and prostate cancer cell proliferation. J Food Sci Tech Mys. (2017) 54:38–44. 10.1007/s13197-016-2426-2 28242901 PMC5305699

[14] Yimit D Hoxur P Amat N Uchikawa K Yamaguchi N. Effects of soybean peptide on immune function, brain function, and neurochemistry in healthy volunteers. Nutrition. (2012) 28:154–9. 10.1016/j.nut.2011.05.008 21872436

[15] Jiang YB Yin QQ Yang YR. Effect of soybean peptides on growth performance, intestinal structure and mucosal immunity of broilers. J Anim Physiol Anim Nutr. (2009) 93:754–60. 10.1111/j.1439-0396.2008.00864.x 19175464

[16] Osho SO Xiao WW Adeola O. Response of broiler chickens to dietary soybean bioactive peptide and coccidia challenge. Poultry Sci. (2019) 98:5669–78. 10.3382/ps/pez346 31247645

[17] Zhuo Y Cao M Li Y Tang L Li WT Jiang XJ. Soybean bioactive peptides supplementation during late gestation and lactation affect the reproductive performance, free amino acid composition in plasma and milk of sows. Livest Sci. (2020) 237:104064. 10.1016/j.livsci.2020.104064

[18] Schwab CG Broderick GA. A 100-year review: protein and amino acid nutrition in dairy cows. J Dairy Sci. (2017) 100:10094–112. 10.3168/jds.2017-13320 29153157

[19] Reynal SM Ipharraguerre IR Lineiro M Brito AF Broderick GA Clark JH. Omasal flow of soluble proteins, peptides, and free amino acids in dairy cows fed diets supplemented with proteins of varying ruminal degradabilities. J Dairy Sci. (2007) 90:1887–903. 10.3168/jds.2006-158 17369230

[20] Jones DF Hoover WH Webster TKM. Effects of concentrations of peptides on microbial metabolism in continuous culture. J anim sci. (1998) 76:611–6. 10.2527/1998.762611x 9498372

[21] Si BW Zhang XD Zhang J Zhang YJ Fu Y Bi YL. Effects of small peptides on milk yield, serum biochemical indices and apparent digestibility of dairy cows. Chin Dairy Cattle. (2019) 9:19–23. 10.19305/j.cnki.11-3009/s.2019.09.004

[22] NRC. Nutrient Requirements of Dairy Catte. 7th ed. Washington, DC: Proceedings of the National Academy of Sciences of the United States of America (2001).

[23] Zheng YH Zhao YY Xue SL Wang W Wang YJ Cao ZJ. Feeding value assessment of substituting cassava (manihot esculenta) residue for concentrate of dairy cows using an *in vitro* gas test. Animals. (2021) 11:307. 10.3390/ani11020307 33530353 PMC7912291

[24] AOAC. Official Methods of Analysis. Gaithersburg, MD: AOAC International (1999).

[25] Vansoest PJ Robertson JB Lewis BA. Methods for dietary fiber, neutral detergent fiber, and nonstarch polysaccharides in relation to animal nutrition. J Dairy Sci. (1991) 74:3583–97. 10.3168/jds.S0022-0302(91)78551-2 1660498

[26] Hao Y Huang S Si J Zhang J Gaowa N Sun X. Effects of paper mulberry silage on the milk production, apparent digestibility, antioxidant capacity, and fecal bacteria composition in holstein dairy cows. Animals. (2020) 10:1152. 10.3390/ani10071152 32645955 PMC7401539

[27] Verdouw H Vanechteld CJA Dekkers EMJ. Ammonia determination based on indophenol formation with sodium salicylate. Water Res. (1978) 12:399–402. 10.1016/0043-1354(78)90107-0

[28] Makkar HPS Sharma OP Dawra RK Negi SS. Simple determination of microbial protein in rumen liquor. J Dairy Sci. (1982) 65:2170–3. 10.3168/jds.S0022-0302(82)82477-6 7153399

[29] Erwin ES Marco GJ Emery EM. Volatile fatty acid analyses of blood and rumeen fluid by gas chromatography. J Dairy Sci. (1961) 44:1768–71. 10.3168/jds.S0022-0302(61)89956-67457884

[30] Bi YL Zeng SQ Zhang R Diao QY Tu Y. Effects of dietary energy levels on rumen bacterial community composition in Holstein heifers under the same forage to concentrate ratio condition. BMC Microbiol. (2018) 18:69. 10.1186/s12866-018-1213-9 29996759 PMC6042446

[31] Chen SF Zhou YQ Chen YR Gu J. Fastp: an ultra-fast all-in-one FASTQ preprocessor. Bioinformatics. (2018) 34:884–90. 10.1093/bioinformatics/bty560 30423086 PMC6129281

[32] Magoc T Salzberg SL. FLASH fast length adjustment of short reads to improve genome assemblies. Bioinformatics. (2011) 27:2957–63. 10.1093/bioinformatics/btr507 21903629 PMC3198573

[33] Caporaso JG Kuczynski J Stombaugh J Bittinger K Bushman FD Costello EK. QIIME allows analysis of high-throughput community sequencing data. Nat Methods. (2010) 7:335–6. 10.1038/nmeth.f.303 20383131 PMC3156573

[34] Haas BJ Gevers D Earl AM Feldgarden M Ward DV Giannoukos G. Chimeric 16S rRNA sequence formation and detection in Sanger and 454-pyrosequenced PCR amplicons. Genome Res. (2011) 21:494–504. 10.1101/gr.112730.110 21212162 PMC3044863

[35] Pruesse E Quast C Knittel K Fuchs BM Ludwig WG Peplies J. SILVA: a comprehensive online resource for quality checked and aligned ribosomal RNA sequence data compatible with ARB. Nucleic Acids Res. (2007) 35:7188–96. 10.1093/nar/gkm864 17947321 PMC2175337

[36] Wang Q Garrity GM Tiedje JM Cole JR. Naive Bayesian classifier for rapid assignment of rRNA sequences into the new bacterial taxonomy. Appl Environ Microb. (2007) 73:5261–7. 10.1128/AEM.00062-07 17586664 PMC1950982

[37] Groot JCJ Cone JW Williams BA Debersaques FMA Lantinga EA. Multiphasic analysis of gas production kinetics for in vitro fermentation of ruminant feeds. Anim Feed Sci Tech. (1996) 64:77–89. 10.1016/S0377-8401(96)01012-7

[38] Parks DH Tyson GW Hugenholtz P Beiko RG. STAMP statistical analysis of taxonomic and functional profiles. Bioinformatics. (2014) 30:3123–4. 10.1093/bioinformatics/btu494 25061070 PMC4609014

[39] Forejtova J Lad F Trinacty J Richter M Gruber L Dolezal P. Comparison of organic matter digestibility determined by *in vivo* and *in vitro* methods. Czech J Anim Sci. (2005) 50:47–53. 10.17221/3994-CJAS18477305

[40] Getachew G Robinson PH DePeters EJ Taylor SJ. Relationships between chemical composition, dry matter degradation and *in vitro* gas production of several ruminant feeds. Anim Feed Sci Tech. (2004) 111:57–71. 10.1016/S0377-8401(03)00217-7

[41] Wang WJ Yang WR Wang Y Song EL Liu XM Wan FC. Effects of soybean small peptides on rumen fermentation and on intestinal and total tract digestion of luxi yellow cattle. Asian-Austral J Anim. (2013) 26:72–81. 10.5713/ajas.2012.12277 25049708 PMC4093062

[42] Tu R Miao J Peng Z Gao Y Bai X Xie X. An *in vitro* study of dietary concentrate: forage ratio and small peptide supplementation effects on ruminal fermentation parameters of yaks. Acta Prataculturae Sinica. (2020) 29:78–88. 10.11686/cyxb2019300

[43] Soto RC Muhammed SA Newbold CJ Stewart CS Wallace RJ. Influence of peptides, amino-acids and urea on microbial activity in the rumen of sheep receiving grass hay and on the growth of rumen bacteria *in vitro*. Anim Feed Sci Tech. (1994) 49:151–61. 10.1016/0377-8401(94)90088-4

[44] Song L Xue B Yin Y Yan T. Effects of tri-peptides consisting of different amino acids on in vitro rumen fermentation parameters of Nanjiang brown goats. Chin J Anim Nutr. (2014) 26:1681–8. 10.1038/s41598-021-03356-y 34916562 PMC8677731

[45] Zhang ZD Wang C Du HS Liu Q Guo G Huo WJ. Effects of sodium selenite and coated sodium selenite on lactation performance, total tract nutrient digestion and rumen fermentation in Holstein dairy cows. Animals. (2020) 14:2091–9. 10.1017/S1751731120000804 32340650

[46] Dechow CD Baumrucker CR Bruckmaier RM Blum JW. Blood plasma traits associated with genetic merit for feed utilization in Holstein cows. J Dairy Sci. (2017) 100:8232–8. 10.3168/jds.2016-12502 28755931

[47] Apelo SIA Bell AL Estes K Ropelewski J de Veth MJ Hanigan MD. Effects of reduced dietary protein and supplemental rumen-protected essential amino acids on the nitrogen efficiency of dairy cows. J Dairy Sci. (2014) 97:5688–99. 10.3168/jds.2013-7833 25022689

[48] Antanaitis R Juozaitiene V Jonike V Baumgartner W Paulauskas A. Milk lactose as a biomarker of subclinical mastitis in dairy cows. Animals. (2021) 11:1736. 10.3390/ani11061736 34200862 PMC8230553

[49] Televicius M Juozaitiene V Malasauskiene D Antanaitis R Rutkauskas A Urbutis M. Inline milk lactose concentration as biomarker of the health status and reproductive success in dairy cows. Agriculture. (2021) 11:38. 10.3390/agriculture11010038

[50] Reynolds CK. Production and metabolic effects of site of starch digestion in dairy cattle. Anim Feed Sci Tech. (2006) 130:78–94. 10.1016/j.anifeedsci.2006.01.019

[51] Nocek JE Tamminga S. Site of digestion of starch in the gastrointestinal-tract of dairy-cows and its effect on milk-yield and composition. J Dairy Sci. (1991) 74:3598–629. 10.3168/jds.S0022-0302(91)78552-4 1744284

[52] Benedet A Manuelian CL Zidi A Penasa M De Marchi M. Invited review: beta-hydroxybutyrate concentration in blood and milk and its associations with cow performance. Animal. (2019) 13:1676–89. 10.1017/S175173111900034X 30854998

[53] Surai PF Kochish II Fisinin VI. Glutathione peroxidases in poultry biology: Part 1. classification and mechanisms of action. World Poultry Sci J. (2018) 74:185–97. 10.1017/S0043933918000284

[54] Fattman CL Schaefer LM Oury TD. Extracellular superoxide dismutase in biology and medicine. Free Radical Bio Med. (2003) 35:236–56. 10.1016/S0891-5849(03)00275-2 12885586

[55] Wang WY De Mejia EG. A new frontier in soy bioactive peptides that may prevent age-related chronic diseases. Compr Rev Food Sci F. (2005) 4:63–78. 10.1111/j.1541-4337.2005.tb00075.x 33430553

[56] Cervenak J Kacskovics I. The neonatal Fc receptor plays a crucial role in the metabolism of IgG in livestock animals. Vet Immunol Immunop. (2009) 128:171–7. 10.1016/j.vetimm.2008.10.300 19027179

[57] Wang Y Nan X Zhao Y Wang Y Jiang L Xiong B. Ruminal degradation of rumen-protected glucose influences the ruminal microbiota and metabolites in early-lactation dairy cows. Appl Environ Microbiol. (2021) 87:e01908–20. 10.1128/AEM.01908-20 33097510 PMC7783353

[58] Ran T Jin L Abeynayake R Saleem AM Zhang X Niu D. Effects of brewers' spent grain protein hydrolysates on gas production, ruminal fermentation characteristics, microbial protein synthesis and microbial community in an artificial rumen fed a high grain diet. J Anim Sci Biotechnol. (2021) 12:1. 10.1186/s40104-020-00531-5 33397465 PMC7780661

[59] Zhao YC Xie B Gao J Zhao GY. Dietary supplementation with sodium sulfate improves rumen fermentation, fiber digestibility, and the plasma metabolome through modulation of rumen bacterial communities in steers. Appl Environ Microb. (2020) 86:e01412–20. 10.1128/AEM.01412-20 32859601 PMC7642074

[60] Niu Q Li P Hao S Zhang Y Kim SW Li H. Dynamic distribution of the gut microbiota and the relationship with apparent crude fiber digestibility and growth stages in pigs. Sci Rep. (2015) 5:9938. 10.1038/srep09938 25898122 PMC4404679

[61] Hao Y Guo C Gong Y Sun X Wang W Wang Y. Rumen fermentation, digestive enzyme activity, and bacteria composition between pre-weaning and post-weaning dairy calves. Animals. (2021) 11:2527. 10.3390/ani11092527 34573493 PMC8467862

[62] Yang ZD Guo YS Huang JS Gao YF Peng F Xu RY. Isomaltulose exhibits prebiotic activity, and modulates gut microbiota, the production of short chain fatty acids, and secondary bile acids in rats. Molecules. (2021) 26:2464. 10.3390/molecules26092464 33922589 PMC8122910

[63] Ricke SC Martin SA Nisbet DJ. Ecology, metabolism, and genetics of ruminal selenomonads. Crit Rev Microbiol. (1996) 22:27–65. 10.3109/10408419609106455 8729959

[64] Wang R Wang M Ungerfeld EM Zhang XM Long DL Mao HX. Nitrate improves ammonia incorporation into rumen microbial protein in lactating dairy cows fed a low-protein diet. J Dairy Sci. (2018) 101:9789–99. 10.3168/jds.2018-14904 30172398

[65] Zhang SH Zeng XF Ren M Mao XB Qiao SY. Novel metabolic and physiological functions of branched chain amino acids: a review. J Anim Sci Biotechno. (2017) 8:10. 10.1186/s40104-016-0139-z 28127425 PMC5260006

